# Effect of CCT137690 on long non-coding RNA expression profiles in MCF-7 and MDA-MB-231 cell lines

**DOI:** 10.17305/bjbms.2019.4155

**Published:** 2020-02

**Authors:** Tuğçe Balcı Okcanoğlu, Çağla Kayabaşı, Cumhur Gündüz

**Affiliations:** 1 Medical Biology Department, Vocational School of Health Services, Near East University, Nicosia, TRNC; 2 Department of Medical Biology, Faculty of Medicine, Ege University, Bornova, Izmir, Turkey

**Keywords:** Breast neoplasms, CCT137690, MCF-7 cells, MDA-MB-231, long non-coding RNA

## Abstract

Long non-coding RNAs (lncRNAs) are involved in a range of biological processes, such as cellular differentiation, migration, apoptosis, invasion, proliferation, and transcriptional regulation. The aberrant expression of lncRNAs plays a significant role in several cancer types. Aurora kinases are increasingly expressed in various malignancies; accordingly, the inhibition of these enzymes may represent a novel approach for the treatment of various cancers. CCT137690, an Aurora kinase inhibitor, displays an anti-proliferative activity in human cancer cell lines. The aim of the present study was to investigate the anti-proliferative and cytotoxic effects of CCT137690 on estrogen receptor (ER)-positive human breast cancer cell line (MCF-7) and ER-negative human breast cancer cell line (MDA-MB-231). In addition, this study was targeted toward determining the changes induced in lncRNA expression levels following the initiation of Aurora kinase inhibitor treatment. The cytotoxic effects of CCT137690 were determined by means of the xCELLigence system. Furthermore, the anti-proliferative role of CCT137690 in breast cancer was investigated by checking the changes in lncRNA expression profiles using quantitative reverse-transcription polymerase chain reaction (qRT-PCR). The half-maximal inhibitory concentrations (IC_50_) of CCT137690 were determined as 4.5 µM (MCF-7) and 7.27 µM (MDA-MB-231). Several oncogenic lncRNAs (e.g., PRINS, HOXA1AS, and NCRMS) were downregulated in both ER-negative and ER-positive cell lines. On the other hand, tumor suppressor lncRNAs (e.g., DGCR5 and IGF2AS) were upregulated in the ER-positive cell line. After CCT137690 treatment, HOXA11AS and PCAT-14 lncRNAs were downregulated in the ER-positive cell lines. In addition, MER11C, SCA8, BC200, HOTAIR, PCAT-1, UCA1, SOX2OT, and HULC lncRNAs were downregulated in the ER-negative cell lines. The results of the present study indicated that Aurora kinase inhibitor CCT137690 could be a potential anti-cancer agent for breast cancer treatment.

## INTRODUCTION

Estrogen receptor (ER) is a nuclear hormone receptor that is involved in the development of breast cancer [1]. Based on the presence of ER, breast cancer can be classified into two groups of ER-negative and ER-positive. According to a large body of evidence, approximately 80% of breast cancers are ER-positive [2]. Aurora kinases entail three isoforms of A, B, and C. Aurora kinases are the most important serine/threonine protein kinases that regulate the function of centrosomes, spindle, and kinetochores for appropriate mitotic progression. According to the literature, Aurora A and B are overexpressed in various malignancies of the breast, colon, nerve tissues, pancreas, and ovary [3,4]. Aurora A and B are expressed in most of the cell types, and their expression is associated with cell proliferation; however, Aurora C expression is limited to germ cells [5].

CCT137690 is a synthetic Aurora kinase inhibitor with high selectivity against all types of Aurora kinases [6]. Inhibition of Aurora kinases is known as a probable therapeutic approach for malignancies. As indicated by the evidence, Aurora kinase inhibitor ZM447439 promotes the adhesion of MDA-MB-231 cells and inhibits the migration of MCF-7 cells [7].

Long non-coding RNAs (lncRNA) transcribed from genomic DNA are a kind of non-coding RNA longer than 200 nucleotides [8,9]. The lncRNAs have an important role in cell proliferation, cell cycle progression, apoptosis, carcinogenesis, and metastasis [10]. They are also defined as the components of oncogenic or tumor suppressor pathways in various types of cancers [11]. While BC200 plays an oncogenic role in human breast cancer tissue [12], IGF2AS reportedly acts as an epigenetic tumor suppressor in human prostate cancer [13]. This type of RNA has the potential to be used as a new cancer biomarker for the development of targeted molecular therapies [14,15].

MCF-7 is a lowly invasive and estrogen-dependent (ER-positive) breast cancer cell line. On the other hand, MDA-MB-231 is a highly invasive, estrogen-independent (ER-negative) cell line [16]. Chena et al. reported that the use of lncRNA HOTAIR could inhibit cell proliferation and induce apoptosis in MCF-7 breast cancer cells [17]. Furthermore, Guan et al. demonstrated that the application of lncRNA FOXD3-AS1 on MDA-MB-231 cell line could result in smaller tumor size and less distant metastasis [18].

Li et al. also demonstrated that lncRNA MIAT is an estrogen-inducible lncRNA and plays an important role in ER-positive breast cancer cell growth. They found that the expression of lncRNA MIAT underwent an increase in MDA-MB-231 (i.e., an ER-negative breast cancer cell line) and S phase [19]. In a review study, Klinge described various lncRNAs and examined their roles in the development of breast cancer. The mentioned researcher highlighted the need for the implementation of quantitative examination and identification of responsible lncRNAs to understand their exact roles and effects [20].

With this background in mind, the current study was conducted to investigate the cytotoxic and anti-proliferative effects of CCT137690 on estrogen-dependent MCF-7 and estrogen-independent MDA-MB-231 breast cancer cells. As another objective, this study was also targeted toward determining the changes in lncRNA expression following the onset of Aurora kinase inhibitor treatment.

## MATERIALS AND METHODS

### Materials

For the purpose of the study, cell culture media (e.g., RPMI-1640 and Leibovitz’s L-15) and fetal bovine serum (FBS) were purchased from the Biological Industries (Kibbutz Beit-Haemek, Israel). CCT137690 (catalog no. S2744) was purchased from Selleckchem and was suspended in dimethylsulfoxide (DMSO). Human breast adenocarcinoma (i.e., MDA-MB-231) and human breast cancer (i.e., MCF-7) cell lines were obtained from the American Type Culture Collection in the ATCC (USA). Furthermore, the RNeasy Mini Kit was supplied from Qiagen, Germany, while the RNAQuant cDNA Synthesis Kit and Disease-Related LncProfiler 96-well Primer Sets were obtained (from the System Biosciences, USA). Additionally, the Maxima SYBR Green qPCR Master Mix was provided by the Thermo Scientific, USA.

### Cell lines

MDA-MB-231 and MCF-7 cell lines were incubated at 37°C with 5% CO_2_ in RPMI-1640 and Leibovitz’s L-15 media, respectively. The given media were supplemented with 1% L-glutamine, 10% inactivated FBS, and 1% penicillin/streptomycin.

### Cytotoxicity assay

Prior to the implementation of the cytotoxicity assay, MCF-7 and MDA-MB-231 cells were subjected to trypsinization. The cells were counted by means of the Cedex XS Analyzer (Roche), then seeded (1×10^4^ cells in 200 µl per well) in triplicate in 96-well E-plates to determine the cytotoxic effects of CCT137690. Subsequently, they were incubated for 24 h before the addition of CCT137690 at a doses range of 50 µM–1.5 µM. The cells were incubated for 48 h, and impedance was monitored every 15 min throughout the period using the xCELLigence system. This system is based on recording the electronic impedance of e-plates, including gold microelectrodes.

The impedance of electron flow caused by adherent cells is reported using a unitless parameter called Cell Index. Cytotoxicity was evaluated by comparing the viability of CCT137690-treated cells with that of untreated control cells using the xCELLigence RTCA software. The calculation of IC_50_ was accomplished by nonlinear regression analysis (Sigmoidal dose-response [variable slope]).

### LncRNA expression profile

#### Total RNA isolation and complementary DNA synthesis

For lncRNA expression profile, total RNA (including small RNAs) was extracted from the CCT137690-treated and untreated MDA-MB-231 and MCF-7 cells (2×10^6^ cells/ml) by means of the RNeasy Mini Kit. Cytotoxic effects (IC_50_) of CCT13769 value were determined for CCT137690 concentration. The concentration and purity of RNA samples were determined by measuring absorbance at the wavelengths of 260/280 and 230/260 nm using the NanoDrop instrument (Thermo Scientific). For further analysis, the RNA samples with the A260/A280 and A230/A260 absorbance ratios of > 2.0 were applied. For complementary DNA (cDNA) synthesis, RNAQuant cDNA Synthesis Kit (System Biosciences) was used according to the manufacturer’s manual.

#### Quantitative reverse-transcription polymerase chain reaction

The Disease-Related lncProfiler Array was used to investigate the anti-proliferative role of lncRNAs in breast cancer. Relative quantitation of 83 lncRNAs was measured by using a Maxima SYBR Green qPCR Master Mix (Thermo Scientific) on LightCycler 480 II (Roche Life Science). In addition, seven human housekeeping genes (i.e., ACTB, B2M, PGK1, GAPDH, HPRT1, RPL1A, and RPL13A) and four small RNA transcript primers (i.e., 7SL scRNA, 5.8S rRNA, U87 scaRNAU6, and smRNA) were applied for normalization.

The quantitative reverse-transcription polymerase chain reaction array plate included one genomic DNA control and one negative control. The relative expression of lncRNAs was determined using the 2^-ΔΔCT^ method. Fold changes of lncRNA expression levels after CCT137690 treatment were evaluated by comparing them with those of the untreated control groups. Log_2_ transformation was applied to the 2^-ΔΔCt^ values of lncRNA expression in the control and CCT137690-treated groups. Fold changes in lncRNA expression and their significance levels were calculated by Student’s t-test in the online software (https://www.qiagen.com/jp/shop/genes-and-pathways/data-analysis-center-overview-page/). In this regard, ±2-fold changes in the expression of lncRNA, compared with those in the control group, were considered statistically significant at a p-value of < 0.05.

## RESULTS

### Cytotoxic effects of CCT137690 on MCF-7 and MDA-MB-231 cells

**FIGURE 1:**
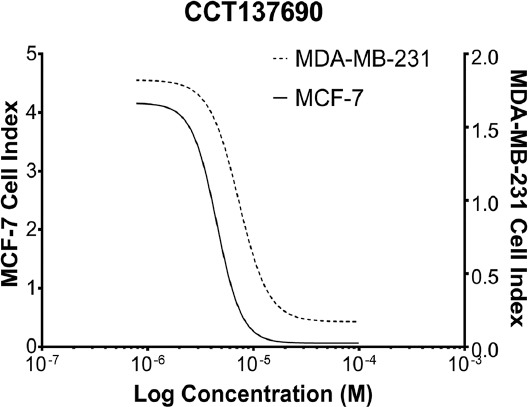
Cytotoxic effects after the implementation of CCT137690 at a concentration range of 50-1.5 µM on MDA-MB-231 and MCF-7 cell lines (IC_50_ dose of CCT137690 in MDA-MB-231 and MCF-7 cell lines after 48 h).

MCF-7 and MDA-MB-231 cells were treated with CCT137690 for 48 h to analyze the cytotoxic effects. The IC_50_ doses of CCT137690 were found to be 4.5 and 7.27 µM for MCF-7 and MDA-MB-231 cell lines (Figure 1) after 48 h by using the xCELLigence system. Untreated MCF-7 and MDA-MB-231 cells were used as the control groups.

### Regulation of lncRNA expressions in both MCF-7 and MDA-MB-231 cells by CCT137690

The changes in the lncRNA expression profiles in MCF-7 and MDA-MB-231 were evaluated after CCT137690 treatment. In the MCF-7 cells treated with 4.5 µM CCT137690, seven lncRNAs (i.e., HOXA1AS, HOXA3AS, HOXA11AS, PCAT-14, PRINS, anti-NOS2A, and NCRMS) were downregulated, and seven lncRNAs (i.e., IGF2AS, CMPD, AKO23948, BC017743, HAR1A, BIC, and DGCR5) were upregulated (*p*<0.05; Table 1).

**TABLE 1:**
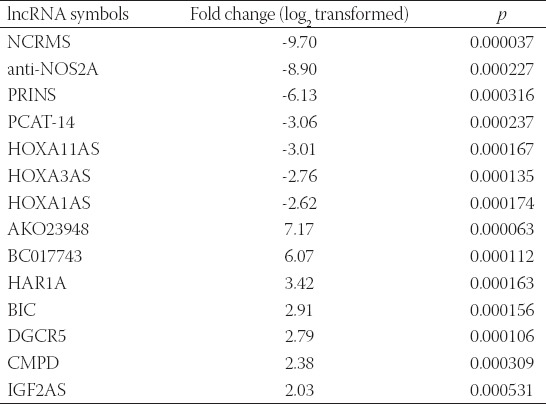
Expression profile of IncRNas In MCF-7 cell line after treatment with 4.5 ΜM CCT137690

Furthermore, the treatment of MDA-MB-231 cells with 7.27 µM CCT137690 resulted in the downregulation of 31 lncRNAs (i.e., UCA1, BC200, 21A, AAA1, HOXA6AS, HOXA1AS, HULC, SOX2OT, SCA8, DLG2AS, DD3, HOTAIR, PRINS, LincRNA-SFMBT2, NCRMS, CCND1 ANCR, L1PA16, ZEB2NAT, BCMS, PSF inhibiting RNA, PCAT-1, MER11C, DISC2, IPW, AKO23948, HAR1B, CMPD, BACE1AS, anti-NOS2A, BC043430, and BC017743) (*p*<0.05; Table 2).

**TABLE 2:**
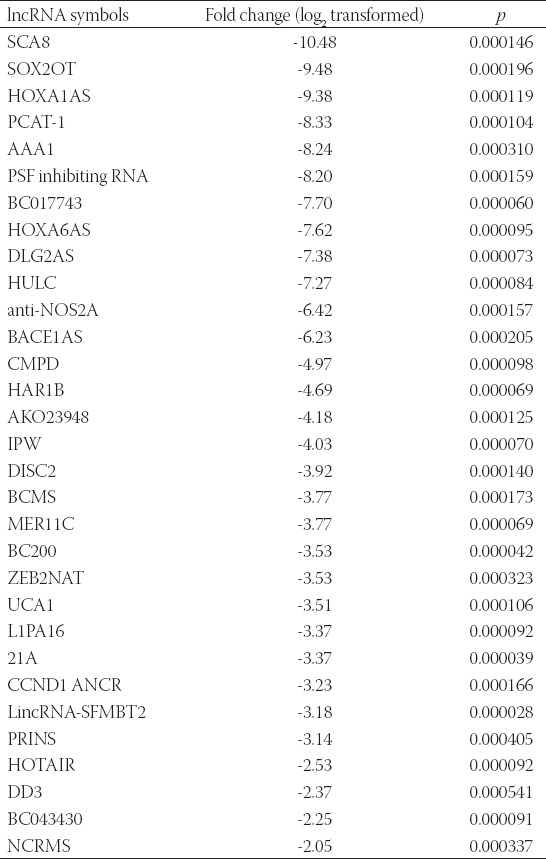
Expression profile of lncrnas in the MDA-MB-231 cell line after treatment with 7.27 µM CCT137690

## DISCUSSION

The lncRNAs are involved in various biological processes, such as cell differentiation and cell cycle control, through transcription, post-transcription, and epigenetic regulation [21]. These RNAs play a role in tumorigenesis and a variety of human malignancies (e.g., papillary thyroid cancer, hepatocellular carcinoma, and breast neoplasms) [21]. The aberrant expression of Aurora kinases can disturb the cell cycle in mitosis and cytokinesis, thereby leading to genetic instability, followed by the stimulation of tumor development. The mechanisms of action of Aurora kinases are based on different isoforms and cancers [7].

According to the literature, HOXA11AS is upregulated in breast cancer cell lines (e.g., SNHG15) and stimulates breast cancer invasion and metastasis by regulating the epithelial-mesenchymal transition. In addition, the knockdown of HOXA11-AS reportedly inhibits the formation of cell colonies and arrests the cell cycle at G0/G1 phase [22]. Lee et al. showed that MER11C, PSF-inhibiting RNA, and SCA8 were also upregulated in the breast cancer cell lines (e.g., SK-BR3) [21].

The results of the present study revealed a 3.01-fold reduction of HOXA11AS gene expression in the ER-positive MCF-7 cell line following CCT137690 treatment. Furthermore, in the ER-negative MDA-MB-231 cell lines, 3.77-, 8.20-, and 10.48-fold decreases were observed in the expression of MER11C, PSF-inhibiting RNA, and SCA8, respectively. In this respect, CCT137690 treatment resulted in the suppression of the oncogenic lncRNAs associated with breast cancer. Regarding this, it can be concluded that CCT137690 has the potential to be applied as an alternative therapy for breast cancer.

The expression level of BC200 lncRNA is associated with tumor grade. BC200 may be an indicator used to assess the progression of a tumor. BC200 is expressed at high levels in the invasive carcinomas of the breast; however, it is not detectable at a significant level in normal breast tissue or benign tumors. Therefore, BC200 can be used as a molecular marker for the diagnosis and treatment of breast cancer [23,24]. In addition, it was indicated that in breast cancer cell lines, the activation of estrogen signaling induces BC200 expression [25].

Based on the evidence, BC200 may be associated with metastasis. Knockdown of BC200 decreases cell migration in cervical carcinoma and breast cancer cell lines [26]. BC200 acts as an oncogene with a negative effect on cell death; in this regard, the silencing of BC200 could induce obvious G0/G1 arrest, cause apoptosis, and reduce invasion in colon cancer cell lines (i.e., HCT-116 and HT29) [27].

In a study conducted by Booy et al., BC200 was found to play a critical role in breast cancer survival. They found that in proliferating cells, the knockdown of BC200 inhibited cell proliferation and induced apoptosis [28]. In our study, we observed a 3.53-fold decrease in the expression of BC200 in the ER-negative cell line after CCT137690 treatment. MDA-MB-231 is a highly aggressive breast cancer cell line, and downregulation of BC200, a molecular marker of invasive breast cancer, may be a valuable outcome for the identification of CCT137690 as a potential breast cancer treatment agent.

In a study conducted on patients with lung cancer, DGCR5 was found to be significantly lower in the neoplastic tissues than in the non-neoplastic tissues. In a lung cancer cell line that was transfected with DGCR5, cell growth, migration, and invasion were reported to significantly decrease [29]. In addition, Liu et al. showed that DGCR5 correlated with better overall survival in lung squamous cell carcinoma [30]. In the same vein, our results showed that CCT137690 treatment upregulates expression lncRNA DGCR5, as a potential tumor suppressor, in MCF-7 cells. As in lung cancer, DGCR5 may also play a tumor-suppressive role in breast cancer cells and contribute to the antiproliferative effect of CCT137690.

HOTAIR expression is increased in both primary and metastatic tumors, and the expression level in primary tumors is a strong indicator of metastasis and mortality. It is suggested that HOTAIR overexpression can be used as a biomarker for the risk of metastasis in patients with ER-positive breast cancer [31]. Tao et al. showed that estrogen promotes HOTAIR expression, and estrogen-induced breast cancer cell migration can be reversed by deleting HOTAIR in the MDA-MB-231 cell line [32]. Similarly, according to our results, the suppression of HOTAIR expression in MDA-MB-231 cells with CCT137690 treatment may play a role in preventing metastasis.

In previous studies on prostate cancer cells, PCAT-1 expression was found to cause functional impairment in homologous recombination with an inhibitory effect on BRCA2 tumor suppressor [33]. Silencing of PCAT-1 reportedly increases the cell proliferation arrest and apoptosis in human bladder cancers [34]. Accordingly, Wen et al. demonstrated that PCAT-1 was upregulated in hepatocellular carcinoma tissue samples, compared to normal tissues. These results suggest that PCAT-1 may have an oncogenic effect on the development of hepatocellular carcinoma [35].

Wang et al. demonstrated the overexpression and association of PCAT-14 with a poor prognosis in patients with hepatocellular carcinoma. Since PCAT-14 promotes proliferation and invasion, in addition to regulating the cell cycle, in hepatocellular carcinoma cells, it has been proposed as a novel prognostic factor and therapeutic target [36]. According to our data, PCAT-1 and PCAT-14, which were decreased as a result of CCT137690 treatment in breast cancer cells, may play a role in the anti-proliferative effects of CCT137690 on breast cancer cells.

In addition to Aurora kinases, CCT137690 is known to inhibit FGFR1 and VEGFR [37]. In a study carried out on hepatocellular carcinoma tissues, a significant positive correlation was found between the expression levels of FGFR1 protein and those of UCA1 [38]. In the current study, UCA1 underwent a decrease after CCT137690 treatment in MDA-MB-231. This decline may be related to CCT137690-mediated FGFR1 inhibition.

Liu et al. demonstrated that UCA1 expression was correlated with a higher stage of breast cancer. Furthermore, they found that the knockdown of UCA1 diminished cell survival and migration ability and promoted apoptosis in vitro [39]. The expression of lncRNA UCA1 was also reported to cause tamoxifen resistance in breast cancer [40]. The decrease in the expression level of this lncRNA, which is critical in breast cancer progression, indicates the promising role of CCT137690 in breast cancer treatment.

SOX20T was found to be upregulated in several cancers, including breast, lung, gastric, and esophageal squamous cell carcinomas [41]. In a study, the MDA-MB-231 cells that ectopically expressed SOX2OT showed significantly slower growth rates than the cells transfected with the control vector [42]. Iranpour et al. studied 38 breast cancer tissues and their adjacent noncancerous tissues and observed a significant overexpression of SOX2OT in tumor tissues, compared with that in the adjacent noncancerous tissues. Therefore, they proposed SOX2OT as an oncogene and demonstrated that SOX2OT-positive breast cancers had a more aggressive clinical course [43]. Accordingly, given the significant reduction of SOX2OT expression (9.48-fold) in the MDA-MB-231 cells treated with CCT137690 in the present study, it can be considered one of the potential novel treatment options for this highly aggressive subtype of breast cancer.

Chen et al. discovered the function of IGF2AS; in this regard, they observed that the overexpression of IGF2AS suppressed prostate cancer cell proliferation and invasion both in vitro and in vivo. Accordingly, they introduced IGF2AS as an epigenetic tumor suppressor [13]. Due to the 2.03-fold increase in IGF2AS in estrogen-dependent breast cancer cells after CCT137690 treatment, we hypothesized that CCT137690 may suppress invasion and metastasis in the early stages of tumorigenesis.

As indicated by a number of studies, lncRNA HULC is highly upregulated in liver cancer [44], functions as an oncogene in epithelial ovarian carcinoma [45], promotes proliferation and inhibits apoptosis in bladder cancer [46], and attenuates the chemosensitivity of hepatocellular carcinoma cells [47]. In the current study, a marked downregulation was observed in HULC expression in MDA-MB-231 cells after CCT137690 treatment. Regarding this, HULC may be concluded to play a role in the cytotoxic effect of CCT137690.

According to the evidence, PRINS acts as an anti-apoptotic lncRNA [48]. In the current study, PRINS lncRNA levels attenuated in both estrogen-dependent and -independent cell lines following CCT137690 exposure. These results are suggestive of the apoptotic effect of CCT137690 on breast cancer cells, in addition to its cytotoxic and anti-proliferative effects. However, this issue should be investigated by further experiments.

Aurora kinases contribute to tumor progression with their mitotic and non-mitotic functions. Aurora-A induces the phosphorylation of such oncoproteins as Akt, mTOR, and p38 MAPK and activates oncogenic signaling pathways [49]. In our investigation, CCT137690 inhibited the activities of aurora kinases, and the subsequent inhibition of the downstream targets of this pathway regulated various lncRNAs in two different breast cancer cell lines. In this regard, two lncRNAs (i.e., HOXA1AS and NCRMS) were down-regulated with CCT137690 in both MCF-7 and MDA-MB-231 cell lines.

Sun et al. compared HOXA1AS expression levels in MCF-7 and MDA-MB-231 cell lines and found that HOXA1AS levels were 9.55-fold higher in MCF-7 cells [50]. In addition, they observed an increase in HOXA1AS levels in the MCF-7 cells treated with 17β-estradiol [50]. Consistent with these findings, in our study, the down-regulation of HOXA1AS in MDA-MB-231 cells after CCT137690 treatment was more prominent, compared to that in MCF-7 cells expressing lower HOXA1AS.

Gu et al. identified that NCRMS levels were elevated in hepatocellular carcinoma as compared to non-cancerous samples [51]. Based on or results, CCT137690 suppressed oncogenic NCRMS expression in both cell lines. Inhibition of Aurora kinase with CCT137690 may lead to the transcriptional regulation of NCRMS by regulating downstream oncogenic pathways and transcription factors. Given that CCT137690 suppressed the transcription of NCRMS more prominently in highly invasive MDA-MB-231 cells than in low-invasive MCF-7 cells, it can be concluded that CCT137690 has the potential to be used for aggressive breast cancers.

The present study facilitated the determination of the anti-proliferative effects of CCT137690 on MCF-7 and MDA-MB-231 cells and changes in lncRNA expression profiles. Consequently, Aurora kinase inhibitor CCT137690 can be suggested as a potential anti-cancer agent for breast cancer treatment.
